# Human papillomavirus vaccination for adult women: Number 6 - June 2022

**DOI:** 10.1055/s-0042-1751331

**Published:** 2022-07-12

**Authors:** Cecília Maria Roteli-Martins, Valentino Magno, André Luis Ferreira Santos, Júlio César Teixeira, Antas Neves Nilma, Susana Cristina Aidé Viviani Fialho

**Affiliations:** 1 Faculdade de Medicina do ABC, Santo André, SP, Brazil; 2 Universidade Federal do Rio Grande do Sul, Porto Alegre, RS, Brazil; 3 Universidade de Taubaté, Taubaté, SP, Brazil; 4 Universidade Estadual de Campinas, Campinas, SP, Brazil; 5 Universidade Federal da Bahia, Salvador, BA, Brazil; 6 Universidade Federal Fluminense, Niterói, RJ, Brazil

## Key points

- Update obstetrician–gynecologists about human papillomavirus (HPV) vaccination for adult women with Febrasgo position about this matter.
- Emphasize there is a generation of women who reached adulthood without HPV vaccination opportunity and highlight benefits provided by vaccine immunization.
- Address epidemiological and immunological aspects of HPV infection and available vaccines.
- Assess the susceptibility of unvaccinated adult women to oncogenic types of HPV and to lesions caused by HPV.
- Assess the difference in susceptibility to recurrence and reinfections after treatment of HPV-associated lesions among vaccinated adult women compared to unvaccinated adult women.
- Provide knowledge of best evidence for HPV vaccination among adult women and collaborate for an updated clinical practice.

## Recommendations

- Consider vaccination even in adult women with previous HPV infection history, as the natural infection does not seem to offer sufficient immunity to prevent new infections occurrence by the same viral type, unlike the immunogenicity induced by HPV vaccines.
- Consider most adult women have negative serological and molecular tests for the viral types included in vaccines.
- Consider second HPV infection peak in the woman’s fifth decade of life.
- Consider adult women without vaccination coverage as still at risk for acquisition of HPV and for developing HPV lesions throughout their lives.
- Consider evidence of recurrences and / or reinfections risk reduction after vaccination of patients with previous lesions caused by HPV and who have already been treated.
- Consider adult women can benefit from individual protection even they are not eligible for vaccination in official programs based on population studies.
- Therefore, we endorse that adult women without HPV vaccination in adolescence, with or without a history of previous infection, may have protection benefits if immunized. In young women (up to 30 years old), these benefits are significant and were demonstrated in several publications. Therefore, this should be part of the medical prescription. However, there is also individual protection for women aged up to 45 years or more who may still be at risk of new infections. This information must be shared by their gynecologists.

## Background

Human papillomavirus (HPV) infection is very prevalent. It occurs early, most of the times after onset of sexual activity. Among diseases associated with HPV infection, cervical cancer stands out. It continues to affect many women worldwide, especially in developing countries like Brazil, even though screening programs are effective when organized. The knowledge about genetic structure of different HPV types and the technological evolution provided vaccines development to prevent infection by oncogenic and non oncogenic HPV types.

Since 2007, HPV vaccines have been administered to adolescents in Immunization Programs around the world. They promote prevention of HPV cervical cancer and clinical lesions. In this target group, mainly between 9 and 12 years old, there is no concerns about vaccine safety and effectiveness. 1 2

In Brazil, the National Program for Immunization (Programa Nacional de Imunizações, or PNI) implemented HPV vaccination in 2014, but vaccination coverage is still low. Currently, there are generations of adult women who have not benefited from HPV vaccination. The aim of this analysis was to present the main scientific evidence in vaccine indication for these women, especially after 25 years old. The PNI covers 9-14-year-old girls. In 2021, the MS/PNI extended the vaccination age for women with HIV, transplant recipients and cancer patients, from 9 to 45 years.(3) The Technical Immunization Advisory Board (CTAI) at a meeting on May 30, 2022, decided to also expand to men with HIV, transplanted and oncological (First meeting of Technical Advisory Board on Immunizations-CTAI, May 30 and 31, 2022, Brasilia DF, oral communication).

As for non-vaccinated young women (up to 26 years old), although they are outside PNI, there is no further scientific discussion, as they have an obvious benefit and vaccination should be indicated. 1 2

## What is the prevalence of HPV infection?

Human Papillomavirus (HPV) infection is the sexually transmitted virus with higher incidence worldwide.

It is estimated that about 600 million people are infected with HPV globally and that 80% of the sexually active population becomes HPV infected during a lifetime. The first peak of incidence occurs during the second decade of life and the second peak during the fifth and sixth decade of life. While the first peak is related to onset of sexual activity, the second can be explained by new exposure or immunity loss. The immunity against HPV infections in adult women is much lower than the immunity against HPV in adolescents. 4 5 6

The relevance of HPV infection was consolidated when its association with cervical cancer was proven. It has been considered as the cause of all cases. The presence of HPV oncogenic DNA was found in 99.7% of cases of cervical cancer. It is the largest cause and effect relationship between an agent and cancer in humans. Several studies have shown that persistent HPV infection is the main risk factor for cervical intraepithelial neoplasia (CIN) and cervical cancer in young and adult females. 4 5 6 7

There is increasing estimate of cervical cancer incidence in countries where there is no organized screening. Despite the availability of vaccines, the last few years estimative are around 530 thousand new cases and 275 thousand deaths annually. In Brazil, the Brazilian National Cancer Institute (INCA) estimates 16,710 new cases for the next years (2020-2022). 4 5 6 7 8

It is important to note that women remain at HPV infection risk throughout their lives, even though the highest contamination rates are in the young population group. Contamination rates are up to 25% over five years in women between 30 and 44 years old. In addition, persistent HPV infection is the main risk factor for CIN and cervical cancer for every age group. 6 7 8 9

## Does immunity develop after natural HPV infection?

Women exposed to HPV infection developed immunity after clearing this infection. This was the subject of several discussions regarding the protective ability for the same viral type. A pioneer study carried out in Costa Rica analyzed 10,049 women. It was observed that the incidence of HPV infection in seropositive women for a certain virus type was similar to seronegative women. This indicate inefficiency of the naturally acquired immunity in protecting against new infection or recurrence. This study evidenced that humoral immunity after natural infection may not prevent new infections, because antibodies levels produced are, generally, low and fall rapidly. They may even be negative. 10 For this reason, adult women previously infected in previous years may not be protected against new infections, including the same viral type.

## What is the clinical value of HPV vaccination for adult women?

Although vaccines were developed for prophylactic use in adolescents before exposure to HPV infections, data show a low adolescent number completing the immunization schedule recommended by PNI in Brazil. On the other hand, studies show that vaccines are effective in adult population even after onset of sexual activity. Besides, a considerable proportion of women did not have active infection due to the types of HPV contained in the vaccine after maturity. 6 7 8 9 10 11 12

Literature also shows evidence of a reduction in relapses with vaccines administration even in patients with previous lesions triggered by HPV and who have already been treated. Although relapses are low (3% -7%), these numbers can decrease by 60% to 80% after vaccination. 13 14 A recent meta-analysis demonstrated the benefits of vaccinating women undergoing excisional treatment for cervical cancer precursor lesions, corroborating some previous studies. 15

About 99.6% of sexually active women up to 45 years old would benefit from HPV vaccines. Studies analyzed the viral infection presence of vaccine virus types in groups of women aged 16 to 23 and 24 to 45 years-old or more women. It was found that most women were seronegative or positive for only one of the viral types studied regardless of age. 11 12 13 14 15 16

The recommended vaccination for women up to 30 years old aims to rescue who have not been properly vaccinated between 9 and 14 years old. The decision must be shared between the physician and 30 to 45-year-old women. It should be recognized that some not adequately vaccinated women may be at risk for new HPV infections and this age group may benefit from vaccination. Logically, the vaccine utility with the coming-of-age will depend on the person’s exposure risk to new infections. The HPV types 6, 11, 16 and 18 vaccine is not licensed for use in women aged > 45 years. 2

A recent cohort study in Sweden showed that HPV vaccination with quadrivalent vaccine was associated with a significant decrease of invasive cervical cancer risk among girls and women aged 10 to 30 at the population level. 17

## Is the vaccine safe in adult female?

There is no record of serious adverse events related to vaccination in any age group. Several regulatory agencies evaluate its safety globally. The HPV vaccines confirm, in practice, an excellent safety profile consistent with initial HPV vaccines trials. Therefore, there is no contraindication to vaccinate women aged up to 45 years or older (depending on the vaccine), since the vaccines are immunogenic and safe for various age groups. They must be individualized for each patient. 2 3 4 5 6 18 19 20

## What are the additional benefits of adult female HPV vaccination?

Besides many adolescents did not receive HPV vaccination at the appropriate time, as discussed, most women seen by gynecologists are above the age limit recommended in the immunization schedule. Therefore, not missing the opportunity to indicate vaccination and avoid HPV infection complications is a fundamental point of the gynecologist care. Even for women who had high-grade precursor lesions and were treated, several studies showed that vaccination after treatment can decrease relapses. It is known that women who have developed HPV lesions have cofactors that facilitate viral action. As these cofactors tend to remain, it may result in pathologies elsewhere. Thus, women with precursor lesions are theoretically at greater risk for other related lesions. In that fashion, vaccination would have a more accurate indication. 12 13 14 19 20

## Does the vaccine confer immunogenicity in adult female?

Antibody response, i.e. immunogenicity in women aged between 24 and 45 years was compared to immunological data of women aged 16 to 23 years, and these were comparable for the HPV-16 type and slightly smaller for HPV-6, 11 and 18. Furthermore, in a study, viral types contained in the vaccine were comparable to those observed at month 48 (end of baseline study), indicating no subsequent reduction in titers between four and six years after vaccination. 12 13 14 15 16 17 18 19 21 22

## Should vaccination in adult women be systematic?

Studies show that vaccination of women aged 30 to 45 years is less effective when compared to vaccination in adolescents and young women (up to 30 years old), especially when DNA-HPV status is ignored or positive. This does not justify the systematic recommendation or calling protocol for vaccination. The woman must be evaluated individually. 17 18 19 20 21 22 23

## Final remarks

The reduction in diseases caused by HPV is causally related to high HPV vaccination coverage among the target groups (children and adolescents). The vaccine is routinely administered before virus exposure.

If universal coverage is available, it will be possible to substantially decrease morbidity and mortality of diseases attributed to HPV worldwide. This could provide a breakthrough in global public health. The school-based vaccination program was suspended years ago. Its return may increase adolescent vaccination coverage. Therefore, it will no longer be necessary to discuss adult female vaccination in the future. Stimulating wide vaccination of adolescents is a fundamental point in primary health care that must not be overlooked. However, we must not miss the opportunity to indicate vaccination for adult women who have not benefited from vaccination as teenagers, especially in countries with lower than expected vaccination rates. This can provide clear benefits as shown by studies. It is important to note that the HPV vaccine administration, at any age, does not replace health promotion actions. Cervical cancer screening should be maintained according to the age group. HPV vaccine and cervical cancer screening are complementary methods to protect women from developing genital cancer. Some fundamental points regarding vaccination still need to be better elucidated among gynecologists and health professionals.

Among them, the indication of vaccination stands out, regardless of whether there is any suspicion or evidence of active HPV infection. In addition, vaccination can be recommended after the treatment of high-grade cervical lesions. It has benefits in reducing relapses, although these are extremely low. Concluding about HPV vaccination in adult women who have not been previously vaccinated, there is no discussion about vaccinating them routinely up to 30 years old, as the benefits have already been demonstrated, and gynecologists should be aware of vaccine prescription. National programs around the world recommend calling these women up for vaccinations. However, data demonstrate there are also benefits in vaccinating women aged up to 45 years or more. These women must be evaluated individually, and the indication must be shared with them. The benefit of vaccinating with the coming-of-age depends on the risk of exposure to new infections.

National Specialty Commission for Vaccines of the Brazilian Federation of Gynecology and Obstetrics Associations (FEBRASGO)

President:

Cecília Maria Roteli Martins

Vice-President:

Nilma Antas Neves

Secretary:

Susana Cristina Aidé Viviani Fialho

Members:

André Luís Ferreira Santos

Angelina Farias Maia

Fabíola Zoppas Fridman

Giuliane Jesus Lajos

Isabella de Assis Martins Ballalai

Juarez Cunha

Júlio Cesar Teixeira

Manoel Afonso Guimarães Gonçalves

Marcia Marly Winck Yamamoto de Medeiros

Renata Robial

Renato de Ávila Kfouri

Valentino Antonio Magno
